# Adjuvant hepatic arterial infusion pump chemotherapy and resection versus resection alone in patients with low-risk resectable colorectal liver metastases – the multicenter randomized controlled PUMP trial

**DOI:** 10.1186/s12885-019-5515-6

**Published:** 2019-04-05

**Authors:** F. E. Buisman, M. Y. V. Homs, D. J. Grünhagen, W. F. Filipe, R. J. Bennink, M. G. H. Besselink, I. H. M. Borel Rinkes, R. C. G. Bruijnen, A. Cercek, M. I. D’Angelica, O. M. van Delden, M. L. Donswijk, L. van Doorn, P. G. Doornebosch, J. Emmering, J. I. Erdmann, N. S. IJzerman, C. Grootscholten, J. Hagendoorn, N. E. Kemeny, T. P. Kingham, E. G. Klompenhouwer, N. F. M. Kok, S. Koolen, K. F. D. Kuhlmann, M. C. Kuiper, M. G. E. Lam, R. H. J. Mathijssen, A. Moelker, E. Oomen-de Hoop, C. J. A. Punt, W. W. te Riele, J. M. L. Roodhart, R. J. Swijnenburg, W. Prevoo, P. J. Tanis, M. Vermaas, M. W. J. Versleijen, F. P. Veuger, M. J. Weterman, C. Verhoef, B. Groot Koerkamp

**Affiliations:** 1 Department of Surgery, Erasmus MC Cancer Institute, Erasmus University, Dr. Molewaterplein 40, 3015 GD Rotterdam, The Netherlands; 2 Department of Medical Oncology, Erasmus MC Cancer Institute, Erasmus University, Rotterdam, The Netherlands; 3 Department of Radiology and Nuclear Medicine, Amsterdam UMC, University of Amsterdam, Rotterdam, The Netherlands; 4 Department of Surgery, Amsterdam UMC, University of Amsterdam, Amsterdam, The Netherlands; 5 Department of Surgery, University Medical Center Utrecht, Utrecht, The Netherlands; 6 Department of Radiology, University Medical Center Utrecht, Utrecht, The Netherlands; 7 Department of Medical Oncology, Memorial Sloan Kettering Cancer Center, New York, USA; 8 Department of Surgery, Memorial Sloan Kettering Cancer Center, New York, USA; 9 Department of Nuclear Medicine, Antoni van Leeuwenhoek, Amsterdam, The Netherlands; 10 Department of Surgery, IJsselland Hospital, Capelle aan den IJssel, The Netherlands; 11 Department of Radiology and Nuclear Medicine, Erasmus MC, Erasmus University, Rotterdam, The Netherlands; 12 Department of Medical Oncology, Antoni van Leeuwenhoek, Amsterdam, The Netherlands; 13 Department of Radiology, Antoni van Leeuwenhoek, Amsterdam, The Netherlands; 14 Department of Surgery, Antoni van Leeuwenhoek, Amsterdam, The Netherlands; 15 Department of Nuclear Medicine, University Medical Center Utrecht, Utrecht, The Netherlands; 16 Department of Medical Oncology, Amsterdam UMC, University of Amsterdam, Amsterdam, The Netherlands; 17 Department of Medical Oncology, University Medical Center Utrecht, Utrecht, The Netherlands

**Keywords:** Colorectal liver metastasis, Resection, Adjuvant chemotherapy, Hepatic arterial infusion, Survival

## Abstract

**Background:**

Recurrences are reported in 70% of all patients after resection of colorectal liver metastases (CRLM), in which half are confined to the liver. Adjuvant hepatic arterial infusion pump (HAIP) chemotherapy aims to reduce the risk of intrahepatic recurrence. A large retrospective propensity score analysis demonstrated that HAIP chemotherapy is particularly effective in patients with low-risk oncological features. The aim of this randomized controlled trial (RCT) --the PUMP trial-- is to investigate the efficacy of adjuvant HAIP chemotherapy in low-risk patients with resectable CRLM.

**Methods:**

This is an open label multicenter RCT. A total of 230 patients with resectable CRLM without extrahepatic disease will be included. Only patients with a clinical risk score (CRS) of 0 to 2 are eligible, meaning: patients are allowed to have no more than two out of five poor prognostic factors (disease-free interval less than 12 months, node-positive colorectal cancer, more than 1 CRLM, largest CRLM more than 5 cm in diameter, serum Carcinoembryonic Antigen above 200 μg/L). Patients randomized to arm A undergo complete resection of CRLM without any adjuvant treatment, which is the standard of care in the Netherlands. Patients in arm B receive an implantable pump at the time of CRLM resection and start adjuvant HAIP chemotherapy 4–12 weeks after surgery, with 6 cycles of floxuridine scheduled. The primary endpoint is progression-free survival (PFS). Secondary endpoints include overall survival, hepatic PFS, safety, quality of life, and cost-effectiveness. Pharmacokinetics of intra-arterial administration of floxuridine will be investigated as well as predictive biomarkers for the efficacy of HAIP chemotherapy. In a side study, the accuracy of CT angiography will be compared to radionuclide scintigraphy to detect extrahepatic perfusion. We hypothesize that adjuvant HAIP chemotherapy leads to improved survival, improved quality of life, and a reduction of costs, compared to resection alone.

**Discussion:**

If this PUMP trial demonstrates that adjuvant HAIP chemotherapy improves survival in low-risk patients, this treatment approach may be implemented in the standard of care of patients with resected CRLM since adjuvant systemic chemotherapy alone has not improved survival.

**Trial registration:**

The PUMP trial is registered in the Netherlands Trial Register (NTR), number: 7493. Date of registration September 23, 2018.

## Background

Colorectal cancer (CRC) is the third most common cancer in the Netherlands. More than half of patients with CRC will eventually develop colorectal liver metastases (CRLM), of whom 25% have resectable disease at first presentation [1]. Most patients develop recurrent disease after curative intent resection of CRLM, which in about 50% of patients is confined to the liver [2]. A large phase III trial investigating perioperative systemic chemotherapy for patients with resectable CRLM found overlapping survival curves: 5-year overall survival (OS) was 51% with perioperative chemotherapy versus 48% with surgery alone (*p* = 0.34) [3, 4]. Therefore, resection without additional chemotherapy is currently the standard of care in the Netherlands and better adjuvant treatment is needed.

The risk of recurrence can be predicted with the clinical risk score (CRS) [5]. The CRS is the sum of five poor prognostic factors: disease-free interval less than 12 months, node-positive CRC, more than one CRLM, largest CRLM over 5 cm in diameter, and serum carcinoembryonic antigen (CEA) level above 200 μg/L. After assigning one point to each of the five risk factors, patients can be stratified into low-risk (0–2 points) and high-risk (3–5 points) of recurrence.

### Hepatic arterial infusion pump chemotherapy

Hepatic arterial infusion pump (HAIP) chemotherapy using floxuridine for liver tumors is a treatment that has been developed at Memorial Sloan Kettering Cancer Center (MSKCC, New York, USA). It is currently not available in the European Union (EU), because floxuridine (FUDR) is not registered in the EU. The biological rationale for intra-arterial treatment is that the hepatic artery rather than the portal vein is responsible for most of the blood supply to liver tumors [6, 7]. Intra-arterial floxuridine is delivered in the hepatic artery via a surgically implantable pump with a catheter in the gastroduodenal artery. Up to 95% of floxuridine is extracted by the liver during the first-pass, allowing an up to 400-fold increase in hepatic exposure with minimal systemic exposure [8, 9]. The pump is filled percutaneously and the liver is continuously perfused with chemotherapy.

Promising results of HAIP chemotherapy have been reported. A randomized controlled trial (RCT) demonstrated superior 2-year overall survival (OS) of 85% in patients with resectable CRLM treated with HAIP and concurrent systemic chemotherapy (5-FU) compared to 69% in patients with resection and systemic chemotherapy (5-FU) only (*p* = 0.02) [10]. A recent retrospective analysis evaluated 2368 consecutive patients undergoing complete resection of CRLM with and without adjuvant HAIP chemotherapy at MSKCC between 1992 and 2012 [11]. The median OS with HAIP chemotherapy was 67 months versus 44 months without HAIP chemotherapy (*p* < 0.001). After adjusting for seven independent prognostic factors in multivariable analysis, the hazard ratio (HR) of HAIP chemotherapy was 0.67 (95% CI: 0.59–0.76, *p* < 0.001) [11]. The median OS in the group without HAIP chemotherapy was similar to the 45 months found in a series of 2715 patients from the UK where no HAIP chemotherapy was used [12]. Subgroup analyses demonstrated that HAIP chemotherapy is particularly effective in low-risk patients (median OS 89 months versus 53 months, *p* < 0.001). In high-risk patients however, the difference in median OS was still statistically significant and clinically relevant, however, less pronounced (50 months versus 37 months, *p* < 0.001) [13].

## Methods/design

### Objective

The primary aim is to compare the progression-free survival (PFS) of surgery with adjuvant HAIP chemotherapy to surgery alone in patients with resectable CRLM with a low CRS 0–2 points). Secondary objectives are to compare OS, postoperative complications, adverse events, quality of life, and costs between the two arms. Pharmacokinetics of intra-arterial administration of floxuridine will be investigated as well as predictive biomarkers for the efficacy of HAIP chemotherapy. In a side study, the accuracy of CT angiography will be compared to radionuclide scintigraphy to detect extrahepatic perfusion.

### Study design

**Fig. 1:**
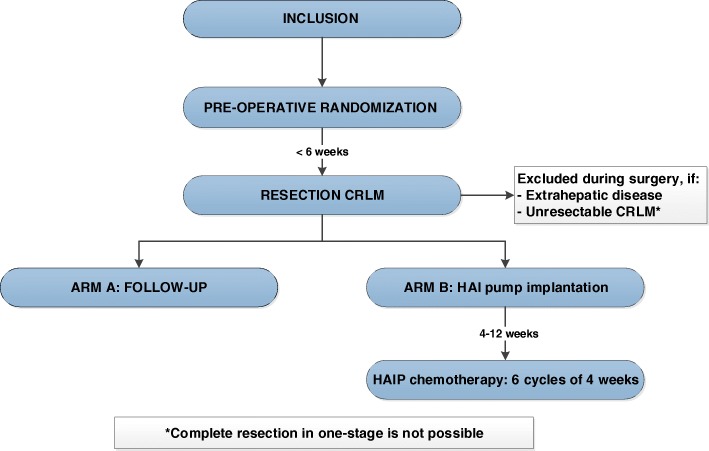
Study flow-chart

The PUMP trial is a phase III randomized controlled open label, multicenter trial to compare the combined efficacy of resection and/or open ablation and adjuvant HAIP chemotherapy to resection and/or open ablation alone in patients with CRC and resectable CRLM with a low CRS (0–2 points). This trial started in August 2018. Five centers participate in this study (Erasmus MC Cancer Institute, Rotterdam; Antoni van Leeuwenhoek, Amsterdam; Academic Medical Center, Amsterdam; Universitary Medical Center Utrecht, Utrecht; IJsselland Hospital, Capelle aan den IJssel). Patients will be randomized in a 1:1 ratio (Fig. 1) to resection of CRLM only (arm A), or resection of CRLM with adjuvant HAIP chemotherapy (arm B). Stratification factors will be center, number of CRLM (< 4 or ≥ 4 CRLM), and size of the largest CRLM (< 5 cm or ≥ 5 cm). Blinding is not feasible because of the nature of the intervention, including a visible subcutaneous pump. In patients who received preoperative chemotherapy for CRLM, the CRS values prior to start of preoperative chemotherapy should be used to determine eligibility. A computed tomography (CT) scan in (early) arterial phase of the liver is required prior to inclusion to determine whether intra-arterial catheter placement is technically possible. The multidisciplinary meeting should determine that complete resection of the CRLM is feasible. Resectability is defined as the opportunity to achieve an R0 resection with a sufficient liver remnant. Randomization will be performed preoperatively if the participant meets all the criteria.

### Study population

Adults with resectable CRLM without extrahepatic disease (EHD) and a low CRS (0–2 points) will be considered for inclusion.

Patients are eligible for this study when they meet the following inclusion criteria:

- age ≥ 18 years;
- ECOG performance status 0 or 1;
- histologically confirmed CRC;
- radiologically confirmed CRLM, amenable for local treatment (resection or open ablation);
- CRS of 0–2. In patients with unknown nodal status of the CRC (in patients with synchronous resection of CRC and CRLM), the nodal status is counted as zero;
- positioning of a catheter for HAIP chemotherapy is technically feasible based on an early arterial phase CT angiography (CTA) (1 mm slide thickness);
- adequate bone marrow, liver, and renal function as assessed by the following laboratory requirements to be conducted within 15 days prior to randomization: absolute neutrophil count (ANC) ≥1.5 × 109/L, platelets ≥100 × 109/L, Hb ≥5.5 mmol/L, total bilirubin ≤1.5 upper normal limit (UNL), ASAT ≤5 x UNL, ALAT ≤5 x UNL, alkaline phosphatase ≤5 x UNL, (calculated) glomerular filtration rate (GFR) >30 mL/min;
- written informed consent.

A potential subject who meets any of the following criteria will be excluded from participation in this study:

- presence of EHD, including positive portal lymph nodes, at the time of liver resection or any time since CRC diagnosis, with exception of small (≤ 1 cm) extrahepatic lesions which are not clearly suspicious of metastases (e.g., pulmonary lesions that are too small to characterize);
- second primary malignancy except in situ carcinoma of the cervix, adequately treated non-melanoma skin cancer, or other malignancy treated at least 5 years prior to inclusion without evidence of recurrence;
- prior hepatic radiation, resection, intra-arterial therapy or ablation;
- CRLM requiring two-staged liver resections;
- liver-first resections; but simultaneous resection of CRC and CRLM is not an exclusion criterion;
- (partial) portal vein thrombosis;
- known DPD-deficiency (heterozygous or homozygous of *DPYP*);
- pregnant or lactating women;
- history of psychiatric disability judged by the investigator to be clinically significant, precluding informed consent or interfering with compliance for HAIP chemotherapy;
- serious concomitant systemic disorders that would compromise the safety of the patient or his/her ability to complete the study, at the discretion of the investigator;
- organ allografts requiring immunosuppressive therapy;
- serious, non-healing wound, ulcer, or bone fracture;
- chronic treatment with corticosteroids;
- serious infections (uncontrolled or requiring treatment);
- participation in another interventional study for CRLM with survival as outcome;
- any psychological, familial, sociological, or geographical condition potentially hampering compliance with the study protocol and follow-up schedule.

## Treatment strategies

### Standard procedures in control arm (arm A)

Patients included in the study should undergo surgery within 6 weeks after signing the informed consent. Local treatment (resection and/or open ablation) of the CRLM in both arms is in accordance with the national guidelines. An intra-operative ultrasound evaluation of the liver will be performed to assure the feasibility of complete resection of the CRLM with an adequate liver remnant. Resection of CRLM can be performed either by minimal-invasive (laparoscopic or robotic) or open approach at the discretion of the surgeon.

### Investigational procedures of the experimental arm (arm B)

**Fig. 2:**
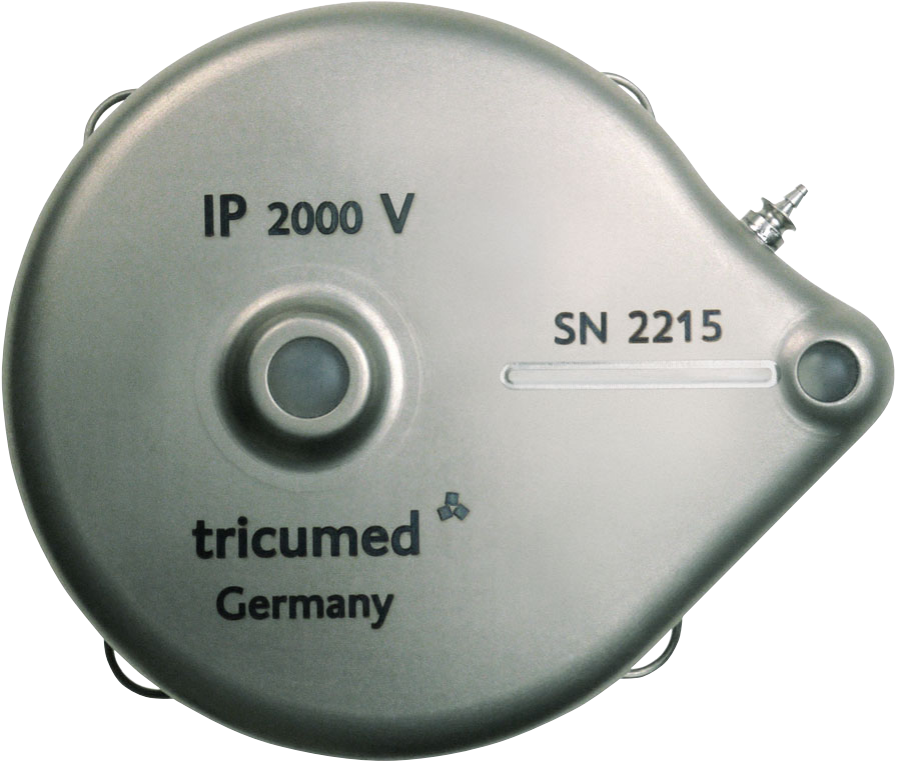
Tricumed infusion pump

**Fig. 3:**
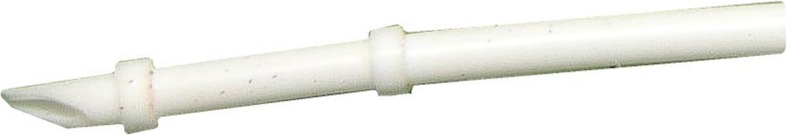
Distal tip of the intra-arterial catheter

The treatment of patients randomized to the experimental arm consists of HAI pump placement following complete resection and/or open ablation of all CRLM. Pump implantation will be cancelled in patients with unexpected unresectable CRLM or EHD detected at the time of surgery. Implantation of the HAI pump (Tricumed IP2000V infusion pump; Fig. 2) is performed by an open or minimal-invasive approach. In patients requiring simultaneous resection of the primary tumor and CRLM, the colorectal resection is performed after pump placement to reduce the risk of pump contamination. The implantation procedure of the infusion pump and dose adjustment protocols have been discussed by previous authors and was optimized for the materials used in this trial [14, 15]. In addition to local treatment of the CRLM, a cholecystectomy is performed to avoid cholecystitis as a result of inadvertent intra-arterial chemotherapy of the gallbladder [16]. The pump catheter is positioned in the gastroduodenal artery (GDA) allowing perfusion of the entire liver without obstructing the flow in the hepatic artery. The pump catheter has rings at the distal end that allow for securing the catheter with non-absorbable ties in the GDA (Fig. 3). In patients with abnormal hepatic arterial anatomy, the GDA is still the preferred site, as long as it connects with a proper hepatic artery perfusing at least one segment of the liver. Perfusion of the entire liver can be achieved in these patients by ligating all accessory and replaced hepatic arteries. Intrahepatic shunts will typically reassure that the catheter perfuses all liver segments.

The entire GDA and the proximal proper hepatic artery are mobilized and dissected circumferentially from their attachments to facilitate insertion of the catheter and to avoid inadvertent perfusion of the pancreas, stomach, or duodenum. Branches to the retroperitoneum arising from the right or left hepatic artery are common and should be ligated. The use of papaverine is optional to gain additional dilatation of the GDA.

Before implantation, a function test of the pump is performed to confirm flow. The pump pocket should be created in the left lower quadrant so that contact with the anterior superior iliac spine and the lower ribs is avoided. The pocket cavity should be 3/4 caudal to the incision to ensure an optimal position of the septum for refills. The catheter is tunneled through the abdominal wall into the abdominal cavity. The pump is secured to the abdominal fascia with nonabsorbable sutures; the catheter should be positioned behind the pump to prevent catheter injury by a needle when accessing the pump percutaneously.

**Fig. 4:**
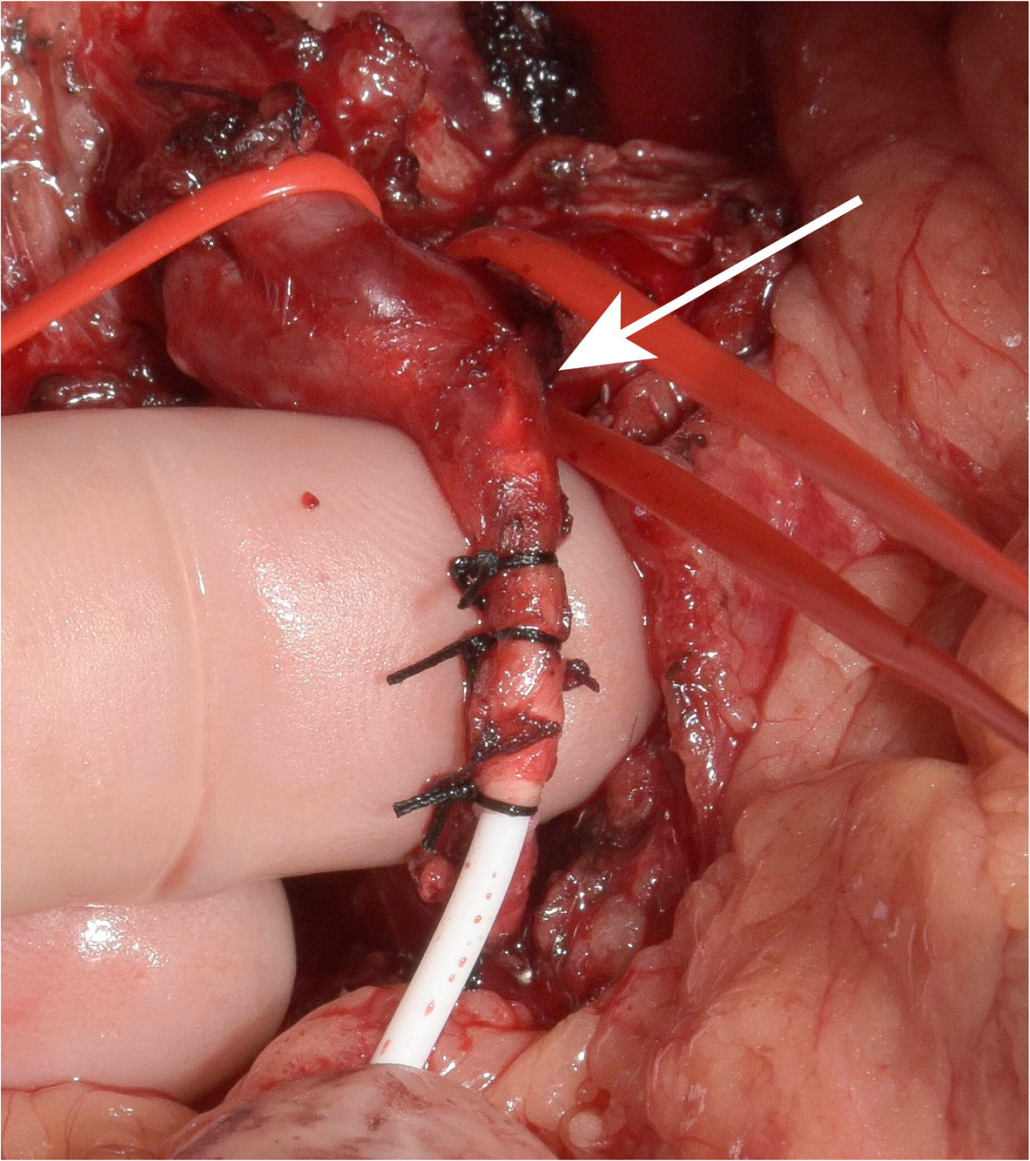
Intra-arterial positioning securing of distal tip of the catheter. Arrow: Tip of the catheter is positioned at the orifice of the GDA

Next, the GDA is ligated with a nonabsorbable tie as far away (at least 2 cm) from the common hepatic artery as possible. Vascular control of the common and proper hepatic arteries is achieved with vascular clamps or vessel loops. Isolated vascular control of the GDA at its orifice can be used alternatively to avoid occlusion of the hepatic artery. A transverse arteriotomy is made in the distal GDA, and the catheter is inserted up to but not beyond the junction with the hepatic artery (Fig. 4). If the catheter protrudes into the common hepatic artery, turbulence of blood flow can lead to increased risk of thrombosis of the hepatic artery. Failure to pass the catheter to the junction leaves a short segment of the GDA exposed to full concentrations of floxuridine without the diluting effect of blood flow, potentially resulting in sclerosis, thrombosis, pseudo-aneurysm with bleeding, or late dislodgment. When positioned, the catheter should be secured with three to four nonabsorbable ties (silk 2.0) proximal to the tying rings on the catheter. Perfusion of both lobes of the liver and lack of extrahepatic perfusion is confirmed by a bolus injection of methylene blue. After the perfusion test, the catheter is flushed with heparinized saline, and the wounds are closed.

### Postoperative procedures experimental arm

Prior to the first administration of intra-arterial chemotherapy, bilobar hepatic perfusion and lack of extrahepatic perfusion are confirmed by:

1. A multiphase or perfusion CT with contrast injection through the bolus port of the pump.
2. Technetium-99-labeled macroaggregated albumin (Tc-99 m MAA) scintigraphy. Tc-99 m MAA is administered through the pump bolus port. Within 1 h after Tc-99 M MAA injection, both planar imaging and a SPECT/CT scan are performed.

Patients with extrahepatic perfusion are evaluated angiographically and aberrant branches embolized with re-testing prior to treatment.

### Drug treatment plan experimental arm

The drug that is used for HAIP is floxuridine (also known as fluorodeoxyuridine (FUDR), Fresenius Kabi, LLC, USA). HAIP chemotherapy with floxuridine has been administered since the early eighties for patients with CRLM in the adjuvant, neo-adjuvant, and induction chemotherapy setting [10, 16–24]. Floxuridine has a half-life of 10 min and the liver extracts 95% of floxuridine during the first pass [8]. Toxic effects have been well characterized. The pump reservoir is filled percutaneously with 0.12 mg/kg floxuridine together with 35,000 U of heparin, 25 mg of dexamethasone, and enough normal saline for a total volume of 35 mL. For patients who are more than 25% above ideal body weight, the actual dose of floxuridine is calculated by using a weight that averages the patient’s actual weight and their ideal weight. Patients will have HAIP administered in a 4-weeks-cycle, with a total of 6 cycles. On day 1, the pump reservoir is filled with floxuridine, dexamethasone, and heparinized saline. On day 15, the pump is emptied and refilled with heparinized saline (35,000 U of heparin and enough normal saline for a total volume of 35 mL) for 2 weeks. Until completion of HAIP chemotherapy, patients will receive a prophylactic proton-pump inhibitor once daily. The use of NSAIDs is discouraged during HAIP treatment. Patients’ complete blood counts and liver tests are monitored every 2 weeks during HAIP chemotherapy. In patients with abnormal liver values, dose reduction or discontinuation of HAIP chemotherapy is performed according to a predetermined protocol (Table 1). Dexamethasone (25 mg) is added to the heparinized saline in case of toxicity according to the values in Table 1 resulting in cessation of floxuridine.

**Table 1:** Dose adjustment schedule

	Reference Value (RV)^a^ Upper limit of normal	% floxuridine dose
Aspartate aminotransferase	2–3 ^a^ RV	80%
	3–4 ^a^ RV	50%
	> 4 ^a^ RV	Hold
Alkaline phosphatase	1.2–1.5 ^a^ RV	50%
	> 1.5 ^a^ RV	Hold
Total bilirubin	1.2–1.5 ^a^ RV	50%
	> 1.5 ^a^ RV	Hold

^a^Reference value is defined as the patient’s value on the first day of the most recent floxuridine dose

## Follow-up

Follow-up for patients both randomized to arm A and arm B will be performed with CEA measurement and abdominal and chest CT including 4-phase liver imaging (year 1–3: every 3 months; year 4–5: every 6 months). The surgical complication score is measured 2 weeks and 3 months after surgery. The chemotherapy toxicity score is measured 2 weeks, three and 6 months after surgery. Quality of life is measured in both arms at baseline, every 3 months in the first year, and 2 and 5 years after surgery.

## Study endpoints and analyses

### Primary endpoint

Primary endpoint of this study will be PFS, calculated from the time between surgery and the first event defined as recurrence or death or last follow-up. Patients still alive without recurrence at last contact are censored.

#### Analysis of the primary endpoint

The formal test for difference in PFS between the two treatment arms will be done with a multivariate Cox regression analysis with adjustment for the stratification factor except hospital. The actuarial method of Kaplan and Meier will be used to estimate survival probabilities, while the Greenwood estimate will be used to construct corresponding 95% confidence intervals (CIs). Kaplan-Meier curves will be generated to illustrate PFS, for all patients as well as by treatment arm. A prespecified subgroup analysis will be performed for the following subgroups: node-negative CRC, CRS of 0 to 1 points, and KRAS wild-type.

### Secondary endpoints

Secondary endpoints include: OS (calculated from surgery until death from any cause; patients still alive at last contact are censored), hepatic PFS, safety, quality of life (EQ-5D + QCQ-C30), and cost-effectiveness. Furthermore, the pharmacokinetic profile of intra-arterial administration of floxuridine will be investigated in more detail. Moreover, we aim to identify predictive biomarkers (circulating tumor DNA) for the efficacy of HAIP chemotherapy. Finally, the accuracy of CT angiography will be evaluated compared to radionuclide scintigraphy to detect extrahepatic perfusion.

## Sample size calculation

A median PFS of 17 months was observed in 228 low-risk patients with resectable CRLM at Erasmus MC treated between 2000 and 2012, without EHD (consistent with arm A). In a multivariable analysis using a consecutive cohort of 779 low-risk patients without EHD, treated with or without HAIP chemotherapy between 2000 and 2012 at MSKCC, a hazard ratio (HR) of 0.60 (95% CI: 0.49–0.75) was found. Given a HR of 0.60 (corresponding to a median PFS of 28 months in arm B), 80% power and a 2-sided significance level α = 0.05, a total of 126 events need to be observed. With an expected accrual rate of 6 patients per month in five centers, 3 years accrual and one additional year of follow-up, and taking into account a drop-out rate of 5%, a total of 230 patients need to be randomized. No interim analysis is planned for survival outcomes.

## Safety analysis

Interim analyses are performed for postoperative complications (grade 3 or higher) and adverse events (serious adverse events plus adverse events of grade 3 or higher) for early detection of unusually high rates of complications and adverse events in the experimental arm (arm B). Interim analyses are planned after inclusion of 20 and 50 patients in arm B.

## Discussion

In this trail patients receive adjuvant HAIP chemotherapy without systemic chemotherapy. HAIP chemotherapy in MSKCC is always combined with concurrent adjuvant systemic chemotherapy. Adjuvant systemic chemotherapy is currently not recommended in Dutch guidelines for patients who underwent complete resection of CRLM, since no difference in OS was found in a large RCT [3, 4]. Some retrospective studies confirmed that adjuvant systemic chemotherapy has no impact on OS in patients with a low CRS [25–27].

A previous RCT from MSKCC, which compared patients who received adjuvant systemic 5-fluorouracil (5-FU) and HAIP chemotherapy with patients who received systemic 5-FU alone demonstrated a beneficial 2-years OS of 85% with HAIP versus 69% with 5-FU alone (*p* = 0.02) [10]. Despite this result, HAIP chemotherapy has not been widely adopted. The NCCN guidelines recommend adjuvant HAIP chemotherapy for CRLM as an option in experienced centers (Category 2B). A retrospective study from MSKCC demonstrated a superior OS of 23 months (67 months versus 44 months) in patients treated with HAIP and concurrent systemic chemotherapy compared to systemic chemotherapy alone in patients with resectable CRLM. These results have renewed interest in HAIP chemotherapy outside MSKCC [28]. Another phase III RCT is required to compare adjuvant HAIP chemotherapy for CRLM with surgery alone. The PUMP trial aims to definitively elucidate the efficacy of adjuvant HAIP chemotherapy in patients with resectable CRLM.

Only low-risk patients without EHD will be eligible for inclusion in the PUMP trial. This subgroup demonstrated to benefit more (median OS 89 months versus 53 months, *p* < 0.001) compared to high-risk patients (median OS 50 months versus 35 months, *p* < 0.001). Furthermore, no survival benefit was found in patients with EHD prior to or at time of resection (median OS 37 months versus 33 months, *p* = 0.92). These results have determined the study design and sample size calculation for the PUMP trial.

HAIP chemotherapy requires a well-trained large multidisciplinary team. A previous RCT investigating intra-arterial chemotherapy for CRLM, performed in 26 centers in Germany, was terminated early due to high complication rates [29]. Therefore, we comprehensively trained and proctored the five multidisciplinary teams participating in the PUMP trial. Moreover, a pilot study prior to the RCT has been conducted to confirm the safety and feasibility.

## Abbreviations

ALAT: Alanine aminotransferase
ANC: Absolute neutrophil count
ASAT: Aspartate aminotransferase
CEA: Carcinoembryonic antigen
CRC: Colorectal cancer
CRLM: Colorectal liver metastases
CRS: Clinical risk score
CT: Computed tomography
ECOG: Eastern cooperative oncology group
EHD: Extrahepatic disease
EU: European Union
GDA: Gastroduodenal artery
GFR: Glomerulo filtration rate
HAIP: Hepatic arterial infusion pump
Hb: Hemoglobin
HR: Hazard ratio
MSKCC: Memorial Sloan Kettering Cancer Center
NCCN: National comprehensive cancer network
OS: Overall survival
PFS: Progression free survival
RCT: Randomized controlled trial
RFA: Radiofrequency ablation
SPECT: Single photon emission computed tomography
Tc-99 m MAA: Technetium-99 m macroaggregated albumin
UNL: Upper normal limit
USA: United States of America
